# The effect of digital storytelling on women's human papilloma virus awareness: Randomized controlled experimental study

**DOI:** 10.1111/jep.14174

**Published:** 2024-10-13

**Authors:** Elif Dağlı, Feyza Aktaş Reyhan, Ahmet Arık

**Affiliations:** ^1^ Department of Health Care Services, Abdi Sütcü Vocational School of Health Services Çukurova University Adana Turkey; ^2^ Department of Midwifery, Faculty of Health Sciences Kütahya Health Sciences University Kutahya Turkey; ^3^ Alparslan Türkeş Secondary School, Adana Provincial Directorate of National Education Adana Turkey

**Keywords:** awareness, digital storytelling, health education, human papilloma virus, woman

## Abstract

**Objective:**

This study aims to determine the effectiveness of the digital storytelling (DS) method in increasing human papilloma virus (HPV) awareness.

**Study Design:**

The study was conducted in the gynecology and obstetrics outpatient clinic of a state hospital between April and December 2023. The universe of the study consisted of women who applied to the outpatient clinic between these dates and volunteered. A total of 80 women who met the inclusion criteria were included in the study (*n* = 40 for each group). Data for the study were gathered using Personal Introduction Form and Human Papilloma Virus Knowledge Scale. Repeated measures ANOVA method was used to determine the difference between the pre‐training, post‐first training and post‐second training measurement scores according to the intervention and control groups. For significant ANOVA results, measurements with differences were compared pairwise using Bonferroni correction. All statistical analyses were examined at the *p* < 0.05 significance level.

**Results:**

Socio‐demographic characteristics and HPV awareness levels of women in the intervention and control groups were similar before training (*p* > 0.05). After the activity conducted with the digital story method in the intervention group, the HPV awareness levels of women in this group increased significantly (*p* < 0.05). Considering the development in each group, an increase in posttest scores is observed in both groups. However, when the increase amounts in each group in the intervention and control groups were examined, it was determined that all awareness levels increased more in the intervention group. In the intergroup comparison, it was determined that the mean measurements of general HPV knowledge, general HPV vaccine knowledge, and knowledge of the current HPV vaccination program of women in the intervention group at the first and second follow‐ups after the training were higher than those in the control group.

**Conclusion:**

The DS method was effective in raising HPV awareness given to the intervention group. Health professionals and researchers can benefit from DS in providing health education and consultancy services such as HPV awareness.

## 1 INTRODUCTION

Cervical cancer is a type of cancer that can be diagnosed and treated early with screening methods.^1^, ^2^ According to WHO (2020) data, it is the 4th most common cancer among women worldwide. It ranks 2nd among women of reproductive age (15–49 years old).^3^ Cervical cancer is closely associated with high rates of morbidity and mortality worldwide.^1^ The high incidence of cervical cancer, inadequate human papilloma virus (HPV) screening and low HPV awareness indicate that HPV infection is common. Increasing public awareness, preventing the occurrence of diseases, and planning and training for protection are important.^3^

HPV is responsible for 4% of all cancers, 99% of cervical squamous cell cancers, 90% of anal cancer, 40% of vulvar cancer, 40% of penile cancer, and 12% of oropharynx cancers.^3^ It accounts for approximately 88% of cervical cancer‐related deaths worldwide. Precancerous lesions of cervical cancer can be prevented with early diagnosis and treatment.^4^ Early diagnosis is very important as these lesions progress to invasive cancers over a period of ~10 years. 80% of those diagnosed with cervical cancer are seen in underdeveloped countries where participation in cervical cancer screening programs is inadequate or not implemented sufficiently.^5^ Globally, an estimated 660,000 new cases and 350,000 women lost their lives to cervical cancer in 2022. In Turkey, it is estimated that 2532 women were diagnosed with cervical cancer in 2020, and approximately 1245 women lost their lives to cervical cancer.^6^, ^7^ HPV is a common disease worldwide and can cause various diseases. These diseases are of great importance in terms of both individual health and the economic burden it creates on health systems.^8^

HPV infections and related diseases affect both women and men. It is estimated that approximately 80% of sexually active women and men will be infected with HPV at least once in their lifetime. In the world, the highest rate of HPV infection is seen especially among young and sexually active women. There are two peaks of infection; the first in young women aged 15–24 and the second in middle‐aged women over 40, suggesting that HPV precautions should be started as early as possible and continued for a long time.^4^, ^5^, ^9^, ^10^

There are studies in the literature with educational interventions to prevent HPV infections and control HPV‐related diseases. These trainings were mostly done through face‐to‐face verbal counseling, printed materials, etc.^11^, ^12^, ^13^ However, today individuals can easily access information on the internet.^14^ Advances in technology are increasing the use of digital media in health professions education. Researchers describe digital storytelling in health education as an evocative, empowering and effective method.^15^ Digital storytelling in health services activates the mindset of the audience with stories, enables the sharing of information and increases the level of learning new content. Essentially, they change behavior by changing motivation. More importantly, storytelling through digital media can lead to a more comprehensive understanding of the content and experiences conveyed.^16^

Digital storytelling combines standalone and first‐person narratives with multimedia (e.g., images, music, narration, animation) to create 3–5 min videos. Digital storytelling shares individuals' lived experiences in a way that traditional storytelling (e.g., oral, written stories) cannot.^17^, ^18^ Individuals can archive digital stories, listen to them again, and distribute them to an endless audience online on websites or through social media. Digital stories can encourage creative and reflective learning in healthcare. By incorporating patient experiences and authentic voices into health education, there is the potential to improve clinician and patient communication. It can also promote humanism and empathy in the delivery of healthcare.^16^ Researchers define digital storytelling in healthcare as an evocative, empowering and effective method.^15^ Digital storytelling is used in a variety of healthcare areas, including mental health, oncology, sexual health promotion, and immigrant health.^15^, ^19^, ^20^, ^21^

Interventions to improve women's health are key to achieving the Sustainable Development Goals. The aim of this study is to evaluate the effect of the digital story method on women's knowledge, beliefs and behaviors about cervical cancer, pap smear test and HPV. In this study, we will compile digital stories from women who have been exposed to HPV, have experienced clinical processes with good and bad prognoses, and have had their children vaccinated against HPV, and women who are of reproductive age, have never had an HPV screening test before, and have a girl child (9–18 years old). We aim to contribute to the HPV national screening program target by reaching women and to increase HPV vaccination rates.

## 2 METHODS

### 2.1 Study design

**Figure 1:**
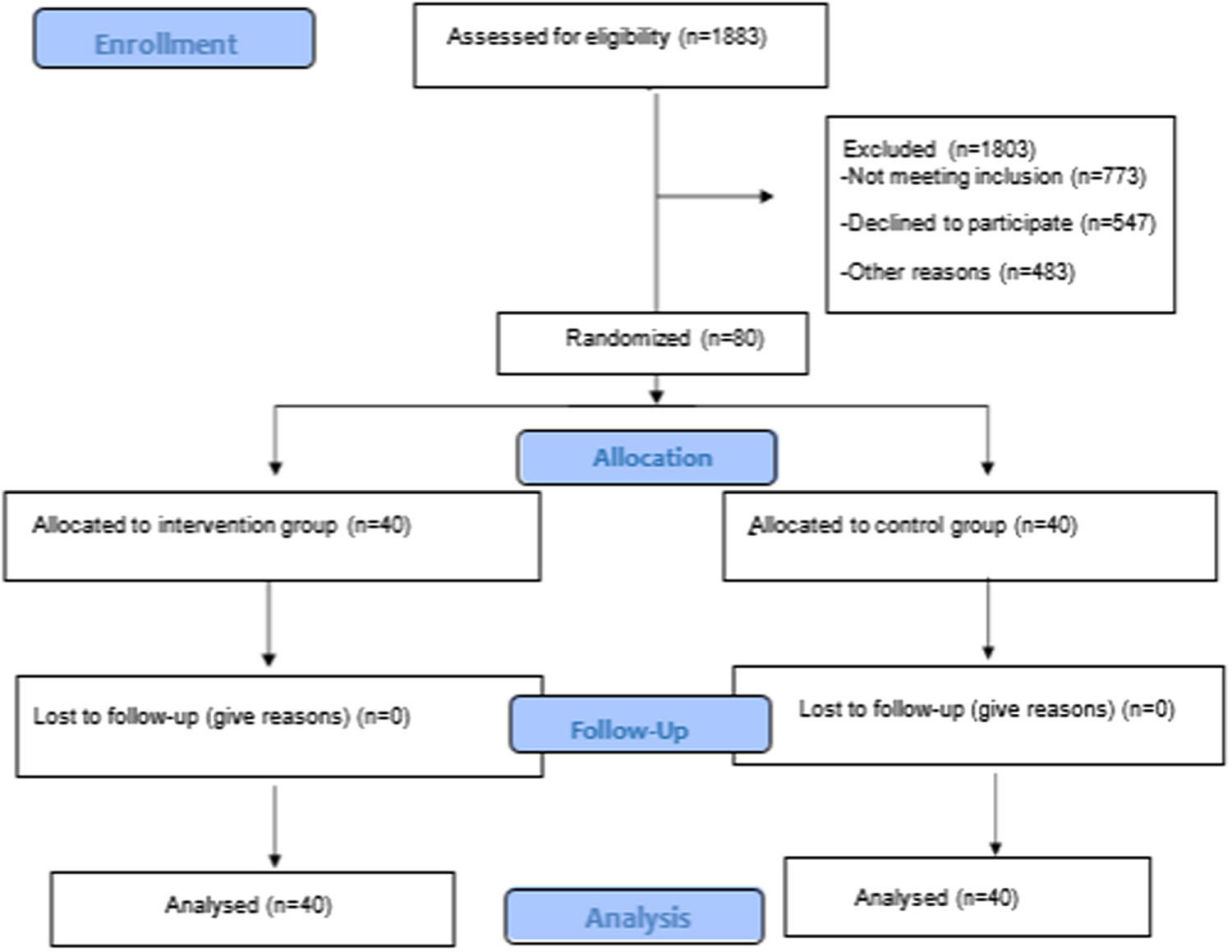
CONSORT flow chart.

This research is a pretest‐posttest randomized controlled experimental study. Made according to CONSORT guidelines (Figure 1).^22^

### 2.2 Setting and samples

The population of the study consisted of women who applied to the gynecology and obstetrics outpatient clinics of state hospital in April and December 2023 for who voluntarily agreed to participate in the study.

G*Power 3.1.9 program was used for sample size calculation and power analysis of the study. In determining the sample size in the study, the COHEN standard effect size was assumed to be 0.70. Thus, while the amount of Type I error was 0.05, the power of the test (power analysis) was 0.80 (α = 0.05, 1‐β = 0.80, effect size = 0.70), and the distribution ratio was minimal according to the one‐to‐one groups distribution ratio.^23^ The sample size was found to be 68 subjects (*n* = 34 for each group). Considering that there would be losses in cases, it was decided to recruit a total of 80 women (*n* = 40 for each group). To determine which group the women who meet the inclusion criteria will be included in the sample, they are randomly distributed to groups through a program (https://www.randomizer.org/) without repeating numbers from 1 to 80; Women will form the intervention and control groups.

#### 2.2.1 Criteria for inclusion in the study

Women who can speak, understand and write Turkish, who are between the ages of 30–49, who have a daughter between the ages of 9–18, who have digital literacy, who have internet access, who have not been screened for HPV before, who have not been vaccinated against HPV, and who volunteer to participate in the research are included in the sample.

### 2.3 Intervention protocol

#### 2.3.1 Intervention group (digital storytelling intervention)

A storytelling HPV video intervention, guided by a situation‐specific theoretical framework and storytelling/narrative communication theory, was developed by the researchers.^24^

#### 2.3.2 Control group

Women in this group were given HPV education within the framework of routine hospital protocol. Storytelling HPV awareness video intervention was also given to the control group after the study.

### 2.4 Data collection

#### 2.4.1 Data collection instruments

Data were collected three times: before the training started, immediately after the training and 1 month after the training.

*Introductory information Form‐IIF*: Developed by researchers in line with the literature.^11^, ^12^, ^13^, ^14^, ^25^ In this form, women were asked about their employment status, income level, education level, number of births, marital status, smoking, family history of cervical cancer, previous sexually transmitted disease, Pap smear test, HPV education, and HPV vaccination of their daughters.

*Human papilloma virus knowledge scale (HPV‐KS):* HPV‐KS was developed by Waller and colleagues in 2013 to measure the knowledge levels of individuals about HPV, HPV vaccines and screening tests.^26^ The scale investigates whether individuals have heard of HPV, HPV vaccines and HPV screening tests before and the extent to which they have knowledge about these subjects. The scale, which has a total of 35 items, includes three sub‐dimensions of 29 items and a six‐item independent sub‐dimension. The first sub‐dimension has 16 items and questions the general knowledge of the participants about HPV. The second sub‐dimension has six items and is related to HPV screening tests. The third sub‐dimension consists of seven items and the participants are asked to answer the items about the HPV vaccine. The participants marked each item of the scale as ‘Yes,’ ‘No’ and ‘I don't know.’ During the evaluation phase, each correct answer is scored as 1, while incorrect answers and ‘I don't know’ statements are scored as 0. The total score is between 0 and 35 and a high score indicates a high level of knowledge about HPV, HPV screening tests and the HPV vaccine.^26^, ^27^

#### 2.4.2 Pilot study

A preliminary application was conducted with 5 women to assess the comprehensibility of the questions in the survey. The women included in the preliminary application were not included in the sample. The number of problematic situations identified in the pilot studies was reduced to zero. The survey was finalized before data collection.

### 2.5 Procedure

#### 2.5.1 Digital storytelling

Digital storytelling is stories made on computers using multimedia components together.^16^ According to another definition; It is the idea of telling a story with strong emotional content by adding images, sound/music, video, etc. via digital multimedia. There are production software such as Movie Maker, iMovie, Photo Story and Corel Video Studio for the production of digital stories.^28^ These production software enrich the content by adding effects, music, and sound to create digital stories.^29^ Digital stories have a preparation process such as writing text, recording sound, combining/editing visuals, adding sound/effects and publishing. With the advancement of technology, most people produce content and share it in different environments.^30^

#### 2.5.2 Preparation of videos

In this research, to create digital stories, scenarios were created from the life stories of 5–10 women who developed cervical cancer due to exposure to HPV. Four videos have been prepared on four topics: diagnosis of HPV and cervical cancer, two different clinical processes related to good and bad prognoses, and prevention and precautions. Each video is approximately 5 min long.

In the 1st video story; The process of a woman exposed to HPV being diagnosed with cervical cancer is mentioned. With this video, information and awareness was created by talking about what HPV is, its epidemiology, risk factors, ways of transmission, associated diseases and cancer.

In the 2nd video story; The clinical course of a woman who was diagnosed with cervical cancer due to HPV exposure and had a good prognosis was mentioned. With this video, awareness was raised about the importance of early diagnosis by talking about cervical cancer signs and symptoms, pap smear test, and the screening program in our country.

In the 3rd video story; The clinical course of a woman who got cervical cancer due to HPV exposure and had a poor prognosis was mentioned. With this video, awareness was raised about the importance of knowledge and early diagnosis by talking about the treatment of cervical cancer, which cannot be diagnosed early and metastasizes.

In the 4th video story; Cervical cancer was diagnosed early by being exposed to HPV, and the process of conveying information about HPV to the people around a woman who survived the disease was mentioned. With this video, information and awareness about protection, precautions and vaccination were created by mentioning the pap smear test and screening program, HPV vaccine administration.

#### 2.5.3 Application of videos to the intervention group

Within the scope of the research, after the first interview with the women to be included in the intervention group in the polyclinic, face‐to‐face pre‐test data were collected with the Introductory Information Form and HPV‐KS from the data collection tools. The contact information of the participants was obtained and digital story videos were sent to them via WhatsApp for them to watch once a week for 4 weeks. The participants' video watching status was monitored and reminders were made via message. The women were contacted again after 4 weeks with the online survey link and the posttest HPV‐KS posttest data were re‐applied.

#### 2.5.4 Providing routine training to the control group

After the first interview with the women in the control group in the outpatient clinic, face‐to‐face pre‐test data were collected with the Introductory Information Form and HPV‐KS from the data collection tools. The contact information of the participants was obtained and the posttest HPV‐KS was re‐applied to the women 1 month later with the online survey link.

The ‘HPV and Cervical Cancer Education Booklet’ was shared as a pdf via WhatsApp with the women included in the control group. The content of the ‘HPV and Cervical Cancer Education Booklet’ was created by the researchers by reviewing the literature.^1^, ^2^, ^7^, ^8^, ^9^, ^10^, ^11^ The topics included in this booklet are: anatomical structure of the female reproductive organs, physiology and HPV, cervical cancer risk factors, screening, treatment, protection methods and HPV vaccine.

### 2.6 Data analysis

Repeated measures ANOVA method was used to determine the difference between the pre‐training, first post‐training and second post‐training measurement scores according to the intervention and control groups. For the significant ANOVA result, the measurements between which the difference was found were compared pairwise using Bonferroni correction. Chi‐square analysis method, a non‐parameric method, was used for the relationship between demographic categorical variables and patient groups. All statistical analyzes were examined at the *p* < 0.05 significance level.

## 3 RESULTS

There is no significant difference between women in the intervention and control groups and the rates of socio‐demographic variables (*p* > 0.05). The rates of women in the intervention and control groups regarding employment status, income level, education level, number of births, marital status, smoking, family history of cervical cancer, previous STI, having a Pap semar test, receiving HPV education, and status of getting HPV vaccination for daughter are similar. The mean ages of women in the intervention (30.05 ± 3.6) and control groups (29.7 ± 4.05) are also similar (Table 1).

**Table 1:** Comparison of demographic characteristics of women in the intervention and control groups.

Variable	Group	Intervention (*N* = 40)	Control (*N* = 40)	Statistics	*p*
		f (%)	f (%)		
Working status	Yes	19 (47.5)	23 (57.5)	χ^2^: 0.802	0.37
	No	21 (52.5)	17 (42.5)		
Income level	Income is less than expenses	20 (50)	23 (57.5)	χ^2^: 0.534	0.766
	Income equals expenses	17 (42.5)	15 (37.5)		
	Income exceeds expenses	3 (7.5)	2 (5)		
Education level	Literate	6 (15)	12 (30)	χ^2^: 2.588	0.274
	Primary education	21 (52.5)	17 (42.5)		
	Secondary education	13 (32.5)	11 (27.5)		
Number of births	Primipar	0 (0)	3 (7.5)	χ^2^: 3.469	0.177
	Multipar	20 (50)	21 (52.5)		
	Grand multipar	20 (50)	16 (40)		
Marital status	Single	11 (27.5)	7 (17.5)	χ^2^: 1.147	0.284
	Married	29 (72.5)	33 (82.5)		
Smoking	Yes	12 (30)	16 (40)	χ^2^: 0.879	0.348
	No	28 (70)	24 (60)		
Family history of cervical cancer	Yes	8 (20)	5 (12.5)	χ^2^: 0.827	0.363
	No	32 (80)	35 (87.5)		
Having had an sexually transmitted ınfection before	Yes	17 (42.5)	22 (55)	χ^2^: 1.251	0.263
	No	23 (57.5)	18 (45)		
Getting a Pap smear test	Yes	6 (15)	10 (25)	χ^2^: 1.25	0.264
	No	34 (85)	30 (75)		
Status of receiving HPV education	Yes	2 (5)	7 (17.5)	χ^2^: 3.13	0.077
	No	38 (95)	33 (82.5)		
Status of getting HPV vaccination for your daughter	Yes	2 (5)	6 (15.0)	χ^2^: 5.12	0.083
	No	38 (95)	34 (85.0)		

Abbreviation: χ^2^, Chi‐square test statistic.

The difference between the first and second post‐training measurements and pre‐training measurements of the women in the intervention group regarding their general HPV knowledge, general HPV vaccine knowledge and knowledge about the current HPV vaccination program sub‐dimension scores is significant (*p* < 0.05). The subscale average scores of women in the intervention group for general HPV knowledge, general HPV vaccine knowledge, and knowledge about the current HPV vaccination program in the first and second measurements after the training were higher than before the training. In addition, the average HPV screening test knowledge subscale score of women in the intervention group in the first measurements after the training was higher than before the training (Table 2).

**Table 2:** Comparison of the pre‐training, first and second post‐training measurements of the human papilloma virus knowledge scale sub‐dimensions in each group.

Group	Scale scores	General HPV information	HPV screening test information	General HPV vaccine information	Information about the current HPV vaccination program
		Mean ± SD	Mean ± SD	Mean ± SD	Mean ± SD
Intervention	Pre‐training^A^	8.53 ± 2.28	4.58 ± 0.9	4.3 ± 0.82	1.75 ± 0.63
	First follow‐up post‐training^B^	11.85 ± 2.02	4.97 ± 0.66	5 ± 0.72	2.43 ± 0.55
	Second follow‐up post‐training^C^	11.33 ± 2.03	4.85 ± 0.77	4.83 ± 0.71	2.35 ± 0.62
	Test statistic	*F*: 38.483	*F*: 10.509	*F*: 8.592	*F*: 18.889
	*p*	*p* < 0.001*	*p* < 0.001*	*p*: 0.001*	*p* < 0.001*
	Difference	B,C > A	B > A	B,C > A	B,C > A
	Effect size	η^2^: 0.669	η^2^: 0.356	η^2^: 0.311	η^2^: 0.499
Control	Pre‐training^A^	8.42 ± 2.27	4.67 ± 0.89	4.33 ± 0.92	1.78 ± 0.73
	First follow‐up post‐training 1^B^	9.34 ± 1.91	4.71 ± 1	4.41 ± 1.17	1.82 ± 0.72
	Second follow‐up post‐training 2^C^	8.45 ± 1.75	4.58 ± 1.04	4.31 ± 1.2	1.78 ± 0.69
	Test statistic	*F*: 10.322	*F*: 2.141	*F*: 2.706	*F*: 1.046
	*p*	*p* < 0.022*	*p*: 0.132	*p*: 0.141	*p*: 0.361
	Difference	B > A			
	Effect size	η^2^: 0.352	η^2^: 0.101	η^2^: 0.103	η^2^: 0.052

*Note*: A: Pre‐training. B: First follow‐up post‐training = immediately after training. C: Second follow‐up post‐training = 1 month after training.

F = Shows the statistical value of the ANOVA method in repeated measurements. Difference: A Bonferroni comparison is made for the significant ANOVA result to show which measurements the difference is between.

Abbreviation: HPV, human papilloma virus.

*p < 0.05.

In the control group, a significant difference was obtained between the general HPV knowledge (*F* = 10.322, *p* < 0.05) subscale score in the women's pre‐training, first and second post‐training measurements. The difference between the general HPV knowledge subscale score of the women in the control group at the first measurement after the training and the mean score before the training is significant (*p* < 0.05). The average general HPV knowledge subscale score of the women in the control group at the first measurement after the training was higher than before the training. However, women in the control group had pre‐training, post‐training first and post‐training second HPV screening knowledge (*F* = 2.141, *p* > 0.05), HPV vaccine knowledge (*F* = 2.706, *p* > 0.05) and knowledge of the current HPV vaccination program (*F* = 1.046, *p* > 0.05) There is no significant difference between subscale scores (Table 2).

There was no significant difference between the pre‐education general HPV knowledge (*t* = 0.64, *p* > 0.05), HPV screening test knowledge (*t* = −0.499, *p* > 0.05), general HPV vaccine knowledge (*t* = −0.128, *p* > 0.05) and knowledge of the current HPV vaccination program (*t* = −0.163, *p* > 0.05) sub‐dimension scores of the women in the intervention and control groups (Table 3).

**Table 3:** Intergroup comparison of measurements of the human papilloma virus knowledge scale sub‐dimensions before training, at the first and second follow‐ups after training.

Group	Scale scores	General HPV information	HPV screening test information	General HPV vaccine information	Information about the current HPV vaccination program
		Mean ± SD	Mean ± SD	Mean ± SD	Mean ± SD
Pre‐training	Intervention	8.53 ± 2.28	4.58 ± 0.9	4.3 ± 0.82	1.75 ± 0.63
	Control	8.42 ± 2.27	4.67 ± 0.89	4.33 ± 0.92	1.78 ± 0.73
	Test statistic	*t*: 0.64	*t*: −0.499	*t*: −0.128	*t*: −0.163
	*p*	*p*: 0.524	*p*: 0.619	*p*: 0.898	*p*: 0.871
	Effect size				
First follow‐up post‐training	Intervention	11.85 ± 2.02	4.97 ± 0.66	5 ± 0.72	2.43 ± 0.55
	Control	9.34 ± 1.91	4.71 ± 1	4.41 ± 1.17	1.82 ± 0.72
	Test statistic	*t*: 6.487	*t*: 1.587	*t*: 4.508	*t*: 4.907
	*p*	*p*: 0.000*	*p*: 0.117	*p*: 0.000*	*p*: 0.000*
	Effect size	η^2^: 0.35		η^2^: 0.207	η^2^: 0.236
Second follow‐up post‐training	Intervention	11.33 ± 2.03	4.85 ± 0.77	4.83 ± 0.71	2.35 ± 0.62
	Control	8.45 ± 1.75	4.58 ± 1.04	4.31 ± 1.2	1.78 ± 0.69
	Test statistic	*t*: 6.315	*t*: 1.349	*t*: 4.3	*t*: 4.581
	*p*	*p*: 0.000*	*p*: 0.181	*p*: 0.000*	*p*: 0.000*
	Effect size	η^2^: 0.338		η^2^: 0.192	η^2^: 0.212

*Note*: First post‐training = immediately after training; Second post‐training = 1 month after training.

*t*: Shows the independent groups t test statistical value.

Abbreviation: HPV, human papilloma virus.

*p < 0.05.

There was no significant difference between the sub‐dimension scores of the women in the intervention and control groups regarding their knowledge of the first HPV screening test after training (*t* = 1.587, *p* > 0.05). However, a significant difference was found between the initial general HPV knowledge (*t* = 6.487, *p* < 0.05), general HPV vaccine knowledge (*t* = 4.508, *p* < 0.05) and knowledge of the current HPV vaccination program (*t *= 4.907, *p* < 0.05) sub‐dimension scores of the women in the intervention and control groups after the training. The post‐education mean initial general HPV knowledge, general HPV vaccine knowledge, and knowledge measures regarding the current HPV vaccination program of women in the intervention group were higher than those in the control group (Table 3).

There was no significant difference between the sub‐dimension scores of the women in the intervention and control groups regarding the second HPV screening test knowledge after training (*t* = 1.349, *p* > 0.05). However, a significant difference was found between the second general HPV knowledge (*t* = 6.315, *p* < 0.05), general HPV vaccine knowledge (*t* = 4.3, *p* < 0.05) and knowledge of the current HPV vaccination program (*t* = 4.581, *p* < 0.05) sub‐dimension scores of the women in the intervention and control groups after the training. The second post‐training general HPV knowledge, general HPV vaccine knowledge, and knowledge measurement averages for the current HPV vaccination program of women in the intervention group were higher than those in the control group (Table 3).

## 4 DISCUSSION

In this study, the effect of the digital story method on women's HPV awareness was investigated. The digital story method was effective in the intervention group. In the study, women's HPV awareness increased positively, and although this increase decreased slightly 1 month after the training, it was still higher than before the training.

Digital storytelling shares individuals' lived experiences in a way that traditional storytelling (e.g., oral, written stories) cannot.^17^, ^18^ Digital stories can encourage creative and reflective learning in healthcare. By incorporating patient experiences and authentic voices into health education, there is the potential to improve clinician and patient communication. It can also promote humanism and empathy in the delivery of healthcare.^16^ In the current study, the subscale mean scores of women in the intervention group for general HPV knowledge, general HPV vaccine knowledge, and knowledge about the current HPV vaccination program in the first and second measurements after the training were higher than before the training. In addition, the HPV screening test knowledge subscale mean score of women in the intervention group in the first measurements after the training was higher than before the training. Although there is increasing interest in the literature on the potential of digital storytelling in the field of health in gaining information and raising awareness in people, only a few studies on HPV screening and vaccination have been reported. Similarly, Kim et al. showed in their study that the storytelling intervention group was twice as likely to have received HPV vaccination compared to the control group.^31^ For experimental studies evaluating HPV vaccine uptake, there are studies reporting higher rates in the narrative HPV video intervention group compared to the information‐based control gblroup.^32^, ^33^ In the current study, for women in the control group who received the hospital's routine HPV awareness training, only the general HPV knowledge subscale mean score was higher than before the training in the first measurement after the training. It was determined that this sub‐dimension score was approximately the same as before the training in the second measurement after the training and that the knowledge was not permanent. Unlike knowledge‐based interventions, storytelling approaches provide information by facilitating identification with storytellers that can prompt participants to change their attitude toward an object while evoking emotions, evoking memories and visual imagination, and encouraging participants to take action on a particular health behavior.^19^, ^34^, ^35^ In the digital age, healthcare professionals need to use innovations to provide quality care to patients.^36^

This study has some strengths and limitations. The strengths of this study include the increase in HPV awareness of the women participating in the study, which contributes to the development of preventive health. In addition, women can store the digital stories in their archives, listen to them again, and distribute them to an infinite number of viewers online via websites or social media. All of these contribute to the increase in HPV awareness. On the other hand, the study has some limitations. The research results are limited to the sample size of the study. The research results can only be generalized within the group where the study was conducted. The research findings are based on the women's self‐reports and were not observed by the researchers. Due to the nature of this study, it was not possible to blind the participants and researchers; however, we provided blinding during data collection and statistical analysis to reduce bias.

## 5 CONCLUSION

In this study, HPV awareness using the digital story method increased positively after the training given, and although this increase decreased slightly 1 month after the training, it was found to be higher than before the training. In the control group, only general HPV vaccine knowledge was higher before the training compared to the first and second measurements after the training. However, HPV screening knowledge, HPV vaccine knowledge, and knowledge of current HPV vaccination program did not change. Digital storytelling has the potential to serve as an effective educational tool in health prevention efforts at a broader level.

Digital storytelling interventions can provide accessible and more equitable information in communities affected by health inequalities. These interventions provide an educational opportunity that is low‐cost, portable, fast, easy, and broadly impactful. It is recommended that studies on HPV awareness training provided by the DH method be conducted in different samples. In future research, visuals could be included in digital stories to help participants better understand medical information.

## CONFLICT OF INTEREST STATEMENT

The authors declare no conflicts of interest.

## ETHICS STATEMENT

Ethical approval for the research was received from Çukurova University Faculty of Medicine Non‐Interventional Clinical Research Ethics Committee Unit (dated 06/01/2023 and numbered 129/86). Permission was received from Adana Provincial Health Directorate (dated 29/03/2023 and numbered E‐96172664‐605.01‐213603768) to conduct the study at Adana City Hospital of the University of Health Sciences. Before starting to collect research data, the purpose of the research was explained to the participants within the scope of the research, and the principle of ‘Informed Consent’ was fulfilled; the principle of ‘Confidentiality and Protection of Confidentiality’ was fulfilled by stating that the information obtained would be kept confidential; and the ethical principles including the principle of ‘Respect for Autonomy’ were fulfilled by accepting those who wanted to participate in the research voluntarily.

## Data Availability

The data that support the findings of this study are available from the corresponding author upon reasonable request.
